# Cross-Linguistic Variation in the Meaning of Quantifiers: Implications for Pragmatic Enrichment

**DOI:** 10.3389/fpsyg.2019.00957

**Published:** 2019-05-15

**Authors:** Penka Stateva, Arthur Stepanov, Viviane Déprez, Ludivine Emma Dupuy, Anne Colette Reboul

**Affiliations:** ^1^Center for Cognitive Science of Language, University of Nova Gorica, Nova Gorica, Slovenia; ^2^2018–2019 EURIAS Fellow at the Collegium – Lyon, Institute for Advanced Studies, University of Lyon, Lyon, France; ^3^UMR5304, Institut des Sciences Cognitives Marc Jeannerod, Bron, France

**Keywords:** quantifier, numerical bound, scalar implicature, R/I-implicature, M-implicature

## Abstract

One of the most studied scales in the literature on scalar implicatures is the quantifier scale. While the truth of *some* is entailed by the truth of *all*, *some* is felicitous only when *all* is false. This opens the possibility that *some* would be felicitous if, e.g., almost all of the objects in the restriction of the quantifier have the property ascribed by the nuclear scope. This prediction from the standard theory of quantifier interpretation clashes with native speakers’ intuitions. In Experiment 1 we report a questionnaire study on the perception of quantifier meanings in English, French, Slovenian, and German which points to a cross-linguistic variation with respect to the perception of numerical bounds of the existential quantifier. In Experiment 2, using a picture choice task, we further examine whether the numerical bound differences correlate with differences in pragmatic interpretations of the quantifier *some* in English and *quelques* in French and interpret the results as supporting our hypothesis that *some* and its cross-linguistic counterparts are subjected to different processes of pragmatic enrichment.

## Introduction

In a broad sense, natural language quantification includes expressions of explicit quantities or numerical proportions (e.g., 50%), as well as a set of expressions that do not directly refer to numbers but express quantities or proportions as more or less vague estimations thereof. Such are the quantificational determiners *some*, *few*, *many*, *half*, *most* (*at least/at most/as many as*) *n* (for a natural number n), *all*, among others. The standard approach in formal semantics that goes back to Barwise and Cooper’s (1981) seminal work, treats these determiners as relations between sets of individuals. In this framework, for instance, the determiner *some*, as in *Some balloons are red*, relates the set of balloons and the set of relevant red objects in a way which requires that the intersection of the two sets is not empty for the sentence to be True in a given situation. Similar semantic definitions are offered for the whole class of other determiners. They are all defined as relations between two sets of individuals. Some examples are given in (1):

(1)     a. [[*some*]] = {<A,B>: A∩B ≠ ∅}
  b. [[*every*]] = {<A,B>: A∩B = ∅}
  c. [[*every*]] = {<A,B>: A⊆B}
  d. [[*most*]] = {<A,B>: |A ∩ B| > ½ |A|}
  e. [[*many*]] = {<A,B>: $\frac{\left|A\cap B\right|}{\left|A\right|}$ > n_c_, for some number n in a context c}

In addition, pragmatic theories which come in some varieties (cf. the classical theory of Grice, 1989; the neo-Gricean theory of Horn, 1984, 2004; Levinson, 2000, the grammatical theory of Chierchia et al., 2012; Chierchia, 2013, the Relevance theory of Sperber and Wilson, 1995) specify a further component (through a different mechanism for each theory) in the meaning of the quantificational expressions that enriches the proposition of which it is part with some pragmatic inference. The most typical example involves enrichment through scalar implicatures. In Horn’s terminology, these implicatures result from (i) the fact that quantifiers are part of a set that forms an entailment scale (see de Carvalho et al., 2016 for evidence of the psychological reality of scales) and as such are always under consideration as possible alternatives and (ii) speakers’ adherence to a pragmatic principle that requires maximal informativeness (Quantity Maxim of Grice/Q-Principle of neo-Griceans) or to the requirements of the exhaustivity operator in the grammatical theory of implicatures. As an illustration we can consider again the example with *some*. The literal meaning of *Some balloons are red* is complemented by a pragmatic inference that *Not all balloons are red* so that the resulting meaning is *Some but not all balloons are red*. The scalar implicature is derived by negating the scalar alternative, *All balloons are red*, to the sentence containing *some* because it is stronger or more informative since it asymmetrically entails the original sentence, but was not chosen by the cooperative speaker. A similar meaning enrichment process applies to all items on the closed quantificational scale which do not occupy its end-points.

However, even if we assume that literal meanings of quantifiers are often strengthened by scalar implicatures, speakers who evaluate the truth of sentences like *Some balloons are red* are expected to always judge as well acceptable the sentence in all contexts in which the size of the set of red balloons relates to the size of the whole set of balloons by a proportion which could be expressed by any number between 0 and 1. That means that situations in which red balloons are 1 or 99% of all balloons are predicted to be just as good as situations in which red balloons are 20% of all balloons in terms of verifying that sentence. This prediction is not always borne out by speakers’ reported intuitions concerning respective contexts. Moreover, according to the standard theories, no cross-linguistic variation is expected in the evaluation of translational equivalents. In other words, quantifiers like *some* or *most* are expected to cover exactly the same range of proportions in different languages.

The goal of this paper is to subject to scrutiny these predictions of the standard semantic-pragmatic treatment of quantifiers. To this end, we report the results of two experiments. Experiment 1 is a cross-language questionnaire study spanning the Germanic, Romance, and Slavic language groups. Two main findings of this experiment are the following: (i) meaning strengthening through scalar implicatures is not sufficient to account for the observed numerical bounds of quantifiers, and (ii) at least the English quantifier *some* is not conceptualized in the same manner cross-linguistically and should not, therefore, receive the same analysis as its counterparts in other languages. In Experiment 2, using a picture-choice test, we further experimentally explore the implications of these results for the theory of scalar implicatures. Specifically, we observe a different pattern of comprehension of sentences containing the English *some* and its French counterpart *quelques*. We interpret the difference as supporting our claim that the meanings of *some* and its crosslinguistic variants result from applying different mechanisms of pragmatic enrichment.

## Quantifiers and Numerical Bounds

### The Psychometric Approach

Quantifier processing has also been in the focus of cognitive psychology. Previous experimental research on the “psychometric” dimension of quantifiers established that the meanings of quantifiers lie on some sort of scale, and suggested that a mapping should hold between a quantifier and its respective range of numerical values (Moxey and Sanford, 2000). Furthermore, the respective numerical range-referring representations of quantifier meanings have been formulated as membership functions used in fuzzy logic, whereby different values pertaining to the quantifier are graded, e.g., between 0, meaning no fit, to 1, implying a perfect fit (Wallsten et al., 1986). For instance, the probability quantifier *likely* might be given a value of 0 for *p* = 0.2, one of 0.1 for *p* = 0.3, and 1.0 for *p* = 0.8. Membership functions encode information about the form of the mapping from an expression to amounts (e.g., variance, skew, kurtosis) as well as central tendency information. These membership functions were found to be stable for a given individual and suggested to be a good substitution for an internalized scale (Wallsten et al., 1993).

However, it was soon recognized that the “psychometric” approach in this form faces serious difficulties, in that that direct assignment of the empirically established range to the respective quantificational expression is very difficult or impossible to implement. Membership functions were found to depend greatly on a number of potentially confounding factors. One such factor is contrast effects that arise because of the within-subject experimental design, whereby subjects are asked to provide values for different quantifiers in a single trial (e.g., Daamen and de Bie, 1991). Another factor has to do with the set size from which proportions are drawn: e.g., low-quantity determiners such as *few* were found to denote a greater proportion when they described small set sizes, compared to larger ones (Newstead et al., 1987). Yet another problem arises from the conflict with base-rate expectations concerning the event described by the quantifier-bearing sentence. For instance, the values assigned to *many* in *Many people enjoyed the party* is higher than in *many doctors are female*, because the former (people enjoying parties), but not the latter, event has a higher base-rate expectation (Moxey and Sanford, 1993). One also faces a serious methodological problem when trying to marry the “psychometric” approach in its present form to the currently standard truth-conditional formal semantics, which interprets sentence meanings in terms of binary truth values 0 and 1. This binary system is in conflict with the rationale behind the membership function allowing an intermediate degree of fit. Irrespective of these shortcomings, it is important to note, however, that the psychometric approach was based on the valid observation that quantifier meanings predicted by the standard semantic-pragmatic approach are not strictly validated by speakers’ intuitions. There is no controversy as to the numerical bounds and set-theoretic meaning of the universal quantifier *every/all* and of the negative one *no* but the rest of the quantifiers apparently need to be reanalyzed.

### The Typicality Approach

The interpretation of quantifiers has recently been reconsidered within a framework based on typicality measures (van Tiel, 2014; van Tiel and Geurts, 2014). This line of research relies on a distinction between typicality and category membership (cf. Fuhrmann, 1991, a.o.). The typicality theory of quantifier interpretation is related to a general mechanism of ascribing typicality differences among members of the same category. One example discussed in van Tiel (2014) regards an experimental study reported in Rosch (1975) where results point to a stable ordering of members of the category BIRD with the robin being evaluated as the most typical in comparison to the rest of the birds denoted by relevant hyponyms of *bird*. In a similar vein, the typicality approach to quantifier interpretation assumes that quantified statements are assigned functions from situations to typicality values. As the authors argue, typicality values can be related to probability values but only if the cardinality of the total set is known. This makes the typicality-based proposal more advantageous than similar proposals of interpreting quantified statements as functions from situations to probability values (cf. Yildirim et al., 2013) since speakers need not necessarily have knowledge about the relevant set cardinality in all situations in which quantifiers are used.

van Tiel and Geurts (2014) investigate typicality judgments associated with the quantifiers *all*, *every*, *few*, *many*, *more than half*, *most*, *some*, *none not all*, *not many* in a large-scale study involving 340 English-speaking participants. They construct visual contexts with 10 black or white circles. The number of black and white circles in each context was manipulated to represent all 11 different possibilities. Using a 7-point Likert scale, participants evaluated the fit between respective quantified sentences and each context. This task was intended to provide typicality judgments. These were contrasted to truth-value judgments which were elicited by using the same material and a task to provide a binary judgment (True/False). The results were interpreted to indicate that typicality judgments were influenced by two factors: set-theoretic definitions and distance from prototype. A necessary condition for a prototype is to be a situation in which the quantified sentence is true according to the respective set-theoretic definition. But, they were also found to depend on competing quantifiers, i.e., a prototypical situation related to a quantifier *q* must be maximally distinct from a prototypical situation related to any competing quantifier *q*′.

Here we focus on three important consequences of the typicality-based analysis of quantifiers. First, the proposal does not make a clear prediction about the interaction between typicality inferences and pragmatic inferences resulting from quantifier alternative competition, i.e., scalar implicatures in non-embedded contexts (see also Cummins, 2014). Second, the proposal leaves no obvious space for cross-linguistic variation. Inasmuch as quantifier numerical bounds are related to prototypes, these are expected to have general cognitive foundations. And finally, if all of the quantifiers in the reported studies involve the same mechanism of association with prototypical values, prototypes should be relatively stable and clearly distinguished even for quantifiers with partially overlapping set-theoretic definitions. This last expectation was not borne out in some cases in the study reported in van Tiel and Geurts. In addition, the claim that prototypes depend on competing quantifiers might need a more detailed formulation given that the study does not distinguish between cases with linguistically provided alternatives and cases with implicitly available alternatives. The last consideration is validated by an experimental study on the processing of two Slovenian counterparts of the determiner *many*, namely *precej* and *veliko* (see Stateva and Stepanov, 2017) and by reported experimental work on processing implicatures within a paradigm that provides alternatives explicitly (cf. Felicity Judgment Task in Foppolo et al., 2012, a.o.).

### Quantifiers as Representations of Proportions: Pezzelle et al. (2018)

The discussion above aimed at motivating the cross-linguistic perspective in studying the perception of quantifiers since potential differences might pinpoint the nature of mechanisms affecting perception. Another important perspective is suggested in Pezzelle et al. (2018), namely the role of proportions as opposed to numerocity in quantifier perception. The study features two experiments, one investigating visually grounded representations and the second one, abstract representations of similarity/difference between quantifiers. Both experiments examine the perception of Italian quantifiers and encompass a list of nine quantifiers including the positive end-point of the proportional scale corresponding to *tutti* (*all*) and the negative end-point corresponding to *nessuno* (*none*). The grounded task used visual stimuli representing a set of objects, part of which were animals in all items. In each trial, the participants were supposed to pick one out of the set of nine quantifiers which best expressed the approximate representation of animals within the whole set of objects. The second experiment asked for metalinguistic judgments about closeness within pairs of quantifiers on a scale from 1 to 7. Both experiments revealed that mental representations of quantifiers represent (non-fixed) proportions rather than cardinalities. The data showed that quantifiers represent an ordered but non-linear scale. Interestingly, the upper part of the scale corresponding to high magnitudes, i.e., *all*, *almost all*, *most*, and *many* involved more overlaps (lower degree of differentiation) in comparison to the lower which was interpreted to indicate a stronger numerical factor in low-magnitude quantifiers. Consequently, the latter type of quantifiers are better differentiated in mental representations.

Using a different protocol we also aim to investigate the mental representations corresponding to quantifiers in four Indo-European languages and compare the results especially to those in Pezzelle et al. (2018). Our main task, however, is to identify the mechanisms behind the different processing patterns.

### The Present Study

We examine the interpretation of quantifiers in two experiments whose aim is to shed further light on a number of relevant questions given the discussion so far. In particular, we aim to identify the main pragmatic factors that influence the processing of quantifiers cross-linguistically. Toward this goal, we address the following questions:

- Is it possible to identify the numerical ranges assigned to different quantifiers and their translational equivalents in other languages? Are numerical ranges encoded in meanings or are they epiphenomenal?
- Are cross-linguistically related quantifiers processed identically? Can we maintain a universal theory of quantifiers on the basis of similarities in the respective numerical values?
- Which pragmatic processes are relevant for the interpretation of quantifiers?
- How are quantifiers with overlapping lexical meanings distinguished?

The main predictions of the present study are rather straightforward. If the classical theory of Barwise and Cooper (1981) and others is on the right track, then, with respect to the quantifier *some*, we should not expect to encounter any specific numerical limitations in the range of evaluated proportions, in English as well as in other tested languages. As pointed out in the Section “Introduction,” given the definition in (1a), situations in which quantified objects constitute between 1 and 99% are predicted to be more or less appropriate for the use of this quantifier. This is not the case for the use of *most* where the definition (1d) restricts the use to the numerical proportions over 50%: therefore, its use in proportions less than 50% should be unacceptable. With respect to quantifier *half*, we obviously expect a peak in acceptability around 50%, while lower and higher proportions should not be acceptable. With respect to *few*, following the standard theory, we view *few* as a negative counterpart of *many* [cf. (1e)] and therefore expect, its upper bound to be well below 50%. In line with neo-Gricean reasoning, we assign *few* to the negative scale <*none*, *hardly any*, *few*> and predict that its lower bound is affected by a scalar implicature negating the two stronger alternatives in the ordered set. Finally, following Penka (2006) which defines *almost* as a member of a Horn-set on a par with *most*, we expect a numerical range for *almost* above that for *most* and excluding the top of the proportional scale.

The predictions concerning the scalar implicature component of the quantifier’s meaning are important in one additional aspect. As both neo-Gricean and Relevance theories predict, meaning strengthening through scalar implicatures should be sufficient to account for the numerical ranges of the quantifier *some* and its crosslinguistic counterparts, that is, the numerical range of *some* must not overlap with numerical range of other quantifiers like *few*, *half*, *most*, or *almost all* if pragmatic enrichment applies.

We were also interested in testing the prediction made by the typicality approach that, inasmuch as quantifier numerical bounds are related to prototypes, the latter are expected to have general cognitive foundations and therefore, no cross-linguistic variation is expected in the meaning of the respective quantifiers, including their numerical ranges.

## Experiment 1

Experiment 1 addresses a similar question to the one of van Tiel and Geurts (2014), namely whether speakers make reference to particular numerical values in their use of different quantifiers. The experimental design is therefore similar to theirs but it, nevertheless, bears some important differences. The main one is that this is a cross-linguistic study involving four languages belonging to different language groups within the Indo-European family: Germanic, Romance, and Slavic. We thus have a possibility to compare how close or different respective lexical counterparts are. The second difference is that we use verbal contexts making reference to a relatively big cardinality of the respective total sets to avoid interference of possible world knowledge.

### Design and Materials

We investigate the cross-linguistic distribution of quantificational determiners by running a series of similarly designed experiments in four languages: English, French, German, and Slovenian. The quantifiers used in the questionnaires per language are listed in Table 1.^1^

**Table 1:** Experiment 1: Quantifiers per language used in target sentences.

English	*few*	*some*	*half*	*most*	*almost (all)*
French	*un peu*	*quelques*	*la moitié*	*la plupart*	*presque (tous)*
German	*wenige*	*einige*	*halbe*	*meisten*	*fast (alle)*
Slovenian		*nekaj*	*polovica*	*veèina*	*skoraj (vse)*


Several clarifications concerning the choice of the target items are in order. First, the reader might wonder why *almost* and its translational equivalents were included in the experimental paradigm given that the classical theory of quantifiers does not normally extend to this determiner. Our decision was partly influenced by a proposal in Penka (2006) based on the argument that *almost* is part of the entailment (Horn-) scale along with determiners like *all* and *most*. If this is the case, then it must belong to that natural class. In addition, we wanted to find out if *almost* acts as an alternative to *most* in forcing it to be restricted to a lower interval than the one predicted by its set-theoretic meaning. Yet another reason for including this item was that we are not familiar with many experimental studies about the numerical bounds of *almost* (cf. Pezzelle et al., 2018).

Second, as Table 1 makes evident, the general cross-language comparison involved five tested quantifiers per language, with one exception: we did not include the Slovenian counterpart of *few*, *malo*, which can be considered a limitation in our design. The appropriate slot in the Slovenian part of the questionnaire was used to test the quantifier *precej* that roughly corresponds to English *many*, instead. All *precej*-sentences were treated as fillers for the purposes of the present study. This quantifier, however, was in the focus of a similarly designed experiment in Stateva and Stepanov (2017); see this work for details.

Third, we did not include in our testing the universal quantifier and the existential quantifier *all* and *no*, because of their extremely narrow-ranged associated proportions, namely 100% in one case and 0% in the other, and therefore trivial (or close to trivial) associated intuitions. We did, however, include the quantifier *half* which is also associated with a fairly trivial proportional range (around 50%) but, because of the more complex actual numerical proportions that we used in this experiment, speakers do not necessarily have a direct access to the result of the respective calculation; as a result, a limited amount of vagueness can also be expected.

**FIGURE 1:**
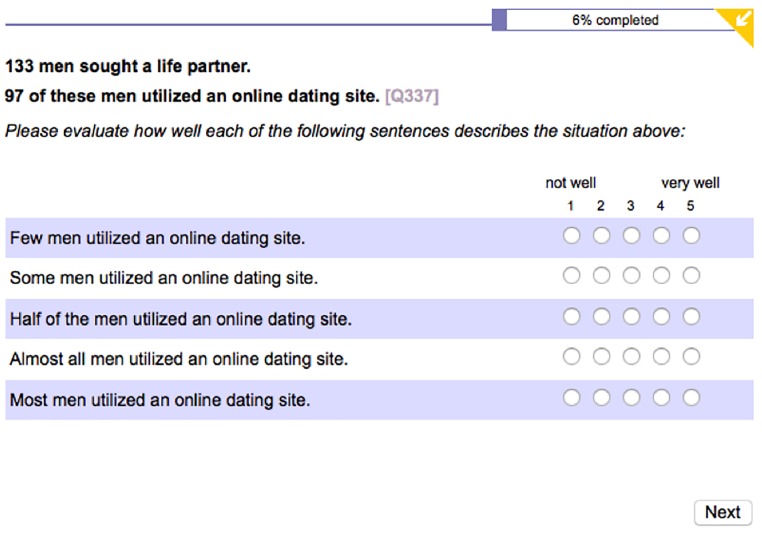
Experiment 1: A sample stimulus screen (the English portion).

Fifty items were prepared as experimental materials. Each item contained a two-sentence context. The first sentence established an event and made reference to the cardinality of a set of individuals. The second sentence referred to one of its subsets. The numbers used in all first sentences of the contexts ranged from 100 to 200. The ratio between first and second number in contexts was manipulated in order for the proportion scale to be covered from 1 to 99% with an increment of 2 within the 50 contexts.^2^ Each context was accompanied by five sentences describing it by using a different quantifier. Furthermore, each sentence was accompanied by a 1–5 Likert scale with annotated end-points *not well* (1) and *very well* (5) in the English, French and Slovenian versions of the questionnaire. German participants had the labels *not well* and *very well* correspond to the scale from 5 to 1, respectively, following a similar convention in the German educational system.^3^ An example of the stimulus materials (from the English portion of the experiment) is given in Figure 1.

### Participants

One hundred eight (24 males) adult self-reported native speakers of English (*N* = 28), French (*N* = 30), German (*N* = 25), and Slovenian (*N* = 25) were recruited for this experiment, and gave an informed consent to participate in the study. The distribution of participants by age groups is shown in Table 2 (participants gave categorical responses regarding their age range in this experiment; the null hypothesis of similar age distribution across different language groups was not confirmed by Fisher’s exact test using Monte Carlo-simulated *p*-value computation: *p* < 0.001).^4^ Approximate age means for each language group were calculated by summing over mid-point of each group range multiplied by frequency of its occurrence and dividing the resulting product by the total number of participants. The English and German participants were undergraduate students at Rutgers University (United States) and the University of Graz (Austria), respectively, and they received course credit. The Slovenian and French participants were students and employees at the University of Nova Gorica (Slovenia) and The University of Lyon (France), respectively. They participated voluntarily and received no compensation. All participants had normal or corrected to normal vision and they were naïve as to the purpose of the study and the research question.

**Table 2:** Experiment 1: Mean times (standard error) spent by the participants on a single screen, broken down by language.

Language	Mean (SE)
English	25.28 (2.32)
French	36.03 (2.35)
German	26.73 (1.65)
Slovenian	30.90 (2.28)


### Procedure

The participants were instructed to read each context carefully and then evaluate, following their first intuition, how well each of the accompanying sentences described the respective context, by clicking on the respective number on the corresponding 5-point scale. All participants received all 50 items in this task. The experiment was administered via the web-based software SoSciSurvey.^5^ The contexts as well as the five target sentences in each context were presented in a pseudo-randomized order for each participant. The participants were allowed to take a break, if necessary, after completing the evaluation of a whole context. Note that this is a task related not only to (semantic) knowledge of quantifier meaning but also a task on pragmatic knowledge of quantifier use. As such, its design involves reasoning, similarly to other tasks targeting pragmatic knowledge.^6^ There were no time limits on finishing the task or evaluation of a particular context. Response times of evaluating all five sentences on each screen were also recorded, mostly for informational purposes (we postpone exploration of the detailed time course in evaluating sentences with specific quantifiers in this type of task for future research; see, e.g., Bott and Noveck, 2004; Hackl, 2009, for relevant discussion).

### Results

Average times spent by the participants on a single screen, broken down by language, are shown in Table 3 (average times less than 7 s and greater than 300 s were trimmed). A one-way ANOVA showed an effect of language on evaluation time (*df* = 3, *F* = 4.95, *p* = 0.003). *Post hoc* pairwise comparison tests (Tukey-type simulations) showed that French was mostly the culprit, with an average evaluation taking about 9 s longer than in German and about 11 s longer than in English. Speakers of the other three languages did not significantly differ in their evaluation time (*df* = 2, *F* = 1.82, *p* = 0.16).

**Table 3:** Experiment 1: Participation by age group and language.

Language/age group	18–20	21–24	25–30	31–35	36–55	Approximate mean
English	9	18	1	0	0	21.6
French	1	17	3	2	7	28.8
German	4	11	6	4	0	24.7
Slovenian	1	3	18	3	0	27.2


For the score analysis, we assumed the mid-scale judgment of 3 points as a threshold for a positive judgment on appropriateness of the respective contexts and excluded datapoints below this threshold. The rationale for not using the set of datapoints collected over the entire set of conditions comes from the perspective seeing quantifiers as markers of numerical proportions. To illustrate the point informally, consider the determiner *half*. It is clear that when an expression such as “half of the dots are red” is evaluated against a finite set of red dots within a particular range, it is only within a very narrow subrange of conditions that this expression will receive high scores, whereas in the vast majority of other cases, it will receive low scores (this was, in fact, the case in our study). Taking the entire set of data points into consideration in this case would lead to the misleading conclusion that speakers generally dislike this determiner, whereas in fact the scores simply reflect the natural situation that the use of this determiner is licensed within a very narrow numerical range. Similar considerations apply in the case of the determiner *all*, as well as for all cardinal quantificational determiners. By analogy, we believe this holds also in the case of the other quantifiers, even though the particular numerical range for this determiner may be hard to establish *a priori* because of their vague character. Thus it would not be appropriate to compare the alleged differences in the use of quantifiers across numerical ranges in which their use is not licensed in principle. In contrast, dividing the Likert acceptability scale in half provides at least a rough estimation of the acceptability boundary. Doing so thus extends the usual tradition of collecting speakers’ evaluations in terms of binary judgments, but also adds the functionality for estimation of the size of the observed differences across different conditions.^7^

**FIGURE 2:**
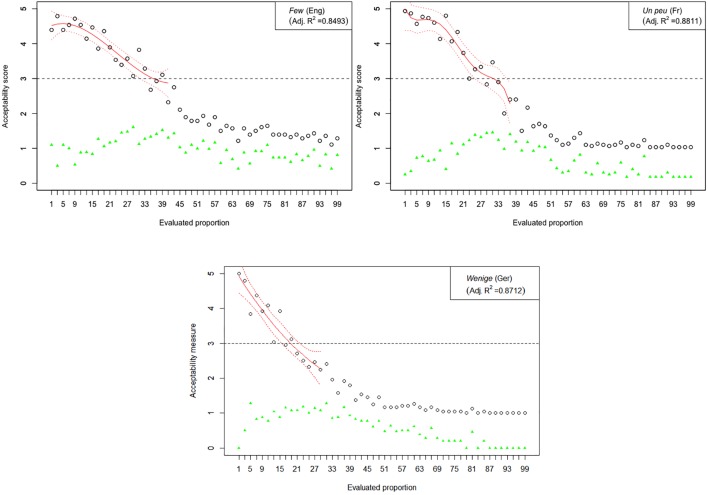
Experiment 1: Mean acceptability scores on *few* and its cross-linguistic variants in the three tested languages (absolute values of standard deviation for respective means are shown in green), together with respective polynomial fit curves and confidence intervals (in red) predicting acceptability in the upper half of the Likert scale.

**FIGURE 3:**
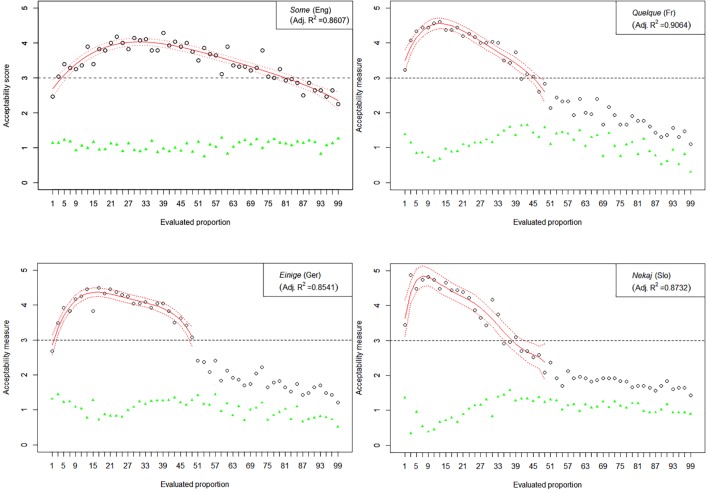
Experiment 1: Mean acceptability scores on *some* and its cross-linguistic variants in the four tested languages (absolute values of standard deviation for respective means are shown in green), together with respective polynomial fit curves and confidence intervals predicting acceptability in the upper half of the Likert scale.

**FIGURE 4:**
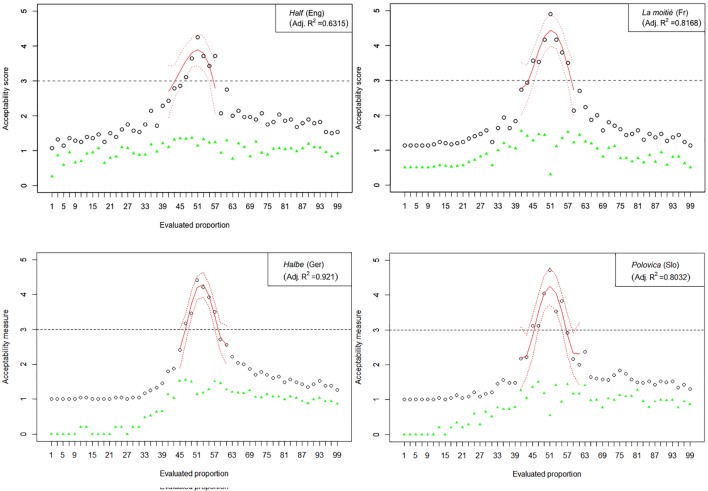
Experiment 1: Mean acceptability scores on *half* and its cross-linguistic variants in the four tested languages (absolute values of standard deviation for respective means are shown in green), together with respective polynomial fit curves and confidence intervals predicting acceptability in the upper half of the Likert scale.

**FIGURE 5:**
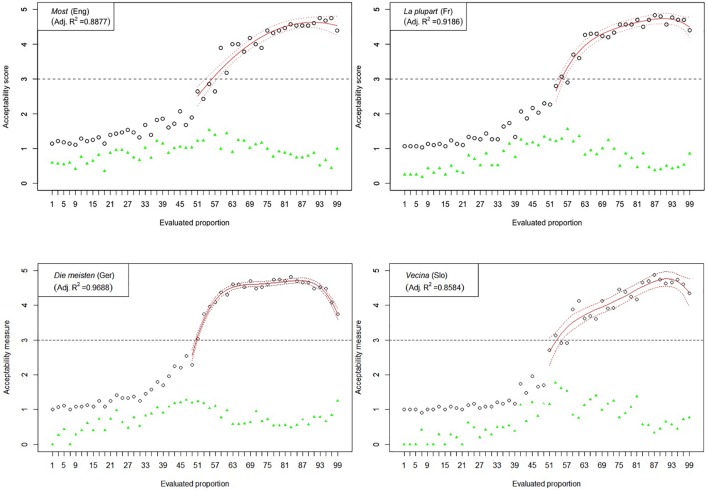
Experiment 1: Mean acceptability scores on *most* and its cross-linguistic variants in the four tested languages (absolute values of standard deviation for respective means are shown in green), together with respective polynomial fit curves and confidence intervals predicting acceptability in the upper half of the Likert scale.

**FIGURE 6:**
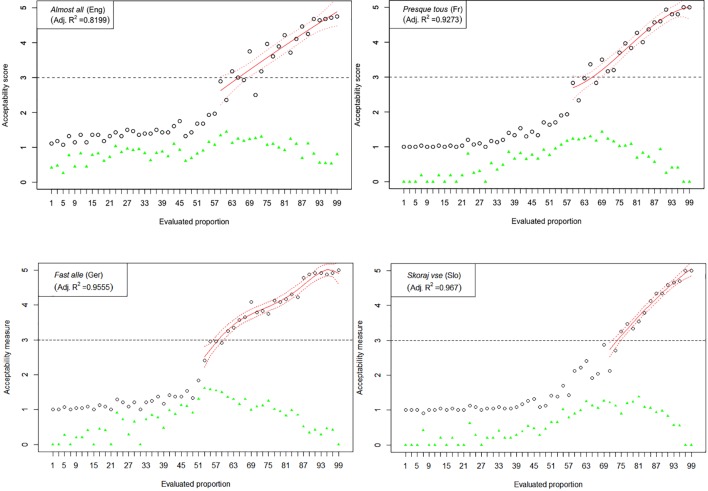
Experiment 1: Mean acceptability scores on *almost all* and its cross-linguistic variants in the four tested languages (absolute values of standard deviation for respective means are shown in green), together with respective polynomial fit curves and confidence intervals predicting acceptability in the upper half of the Likert scale.

The results of Experiment 1 are graphically represented in Figure 2–6. The graphs in the figures summarize acceptability scores in the upper half of the Likert scale per language and per respective quantifier together with respective polynomial fit curves and confidence intervals. Regression models were used to fit the data using polynomial functions. As can be seen from the figures, different quantifiers were judged acceptable in different ranges of proportions. In particular, the numerical proportions characterized by respective cross-linguistic counterparts of *few* appear to be restricted well below 50%, with the score peaking in the first quarter (<25%) of the proportional range. On the other hand, *most* and *almost all* are predictably evaluated higher with proportions of 50% and above. The scores on *most* tend to a plateau in the upper part of the numerical range (>50%), whereas the acceptability on *almost all* increases more steeply toward the last quarter (>75%). The determiner *half* received most of the acceptable scores midrange, peaking around 50% and sharply dropping before and after that.

The results of Experiment 1 revealed that, despite the relatively small sample sizes in this experiment, speakers of all four languages follow consistently similar patterns of evaluating the quantifiers with the meaning of *few*, *half*, *most*, and *almost all*. An important exception in this picture concerns the English quantifier *some* (Figure 3). The numerical proportions whose characterization by non-English counterparts of *some* was acceptable ranged from 3% to slightly less than 50% of the total number of items in the three languages under consideration, namely, German, French, and Slovenian. In contrast, English speakers found proportions in the range between 3 and about 80% of the total number of items at issue, as acceptable to be characterized by *some*. In other words, the range of proportions that can be characterized by the meaning of *some* is 60% larger in English than in the other three tested languages.

**FIGURE 7:**
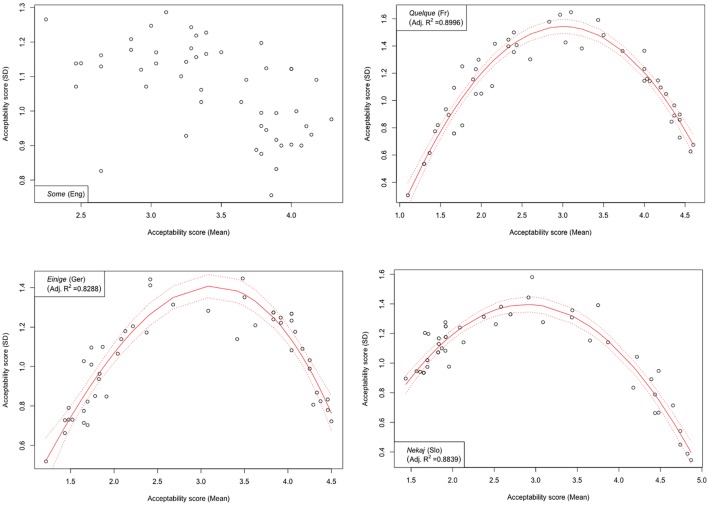
Experiment 1: Standard deviation in the collected score data vs. mean score (both in units on a Likert scale). Each graph represents the results on quantifier *some* and its cross-linguistic variants. Also shown are best (quadratic) fit lines, confidence interval, and the coefficient of multiple determination *R*^2^.

Another notable anomaly pertaining to the English quantifier *some* that we observed in contrast with its cross-linguistic counterparts concerns the correlation of mean score values with respective pooled variance. Mean scores and respective measures of variance such as standard deviations were previously shown to be inherently correlated in studies using Likert scales, whereby standard deviations tend to be smaller if mean values are closer to the extreme points of the Likert scale and increase toward the middle. This trend, when observed over the entire evaluation, can be described with a quadratic regression model and graphically represented by a parabola with a peak around mid-range (Lipovetsky, 2017). In our study, all except one of the tested quantifiers demonstrated a reliable quadratic trend, peaking around the mid-scale (3) and declining on both ends (1 and 5).^8^ The only exception was indeed English *some*, where no discernible trend could be identified, suggesting that means and standard deviations are not correlated here (adjusted *R*^2^ < 0.27). This state of affairs is depicted in Figure 7, in which the results from the English *some* are contrasted with its cross-linguistic counterparts; the latter also serve as representative examples of the polynomial trend observed in the evaluations of the other tested quantifiers.

### Discussion

The reported study demonstrated a lot of unanimous decisions of quantifier evaluation across languages. In most cases it looks like participants intuitively follow a similar mechanism of rough estimation of the proportions and match the outcome against a given quantifier. We can hypothesize that if numerical bounds are related to these determiners are stable, these bounds have a universal character (as much as such a generalization is warranted with observations from a limited language sample of four languages).

However, we have also discovered a divergence of behavioral responses with respect to the determiner meaning *some*. The divergence is twofold: (i) the range of proportions that can be characterized by the meaning of *some* is 60% larger in English than in the other three tested languages; and (ii) the mean scores received from evaluating sentences with English *some* did not correlate with respective standard deviations, in contrast with its respective cross-linguistic counterparts. We take these results to indicate that English speakers are likely to treat this determiner differently compared to speakers of the other tested languages. In particular, the first observation suggests that one way in which this can be different is that English speakers do not associate *some* with the same numerical range as speakers of the other languages.

As concerns the second difference, we tentatively suggest that a reduced standard deviation at the extreme ends of a Likert scale indicates a greater speakers’ confidence or certainty in their judgments, while intermediate scorings are more volatile. Thus a standard deviation can be seen as a measure at the continuum between less and more confident judgments. Both speakers’ confidence and volatility should be seen as qualities at the population, rather than individual, level. At the individual level, the ability to give a “confident” judgment in either direction is a function of a well-defined task. One will not be able to produce a confident judgment if the task conditions are in some sense vague, or allow for more than one “correct” answer, to the speaker. We will argue that this is precisely the case with English *some*, whereby our context conditions allowed for interpreting *some* in more than one way, differently from the way speakers of the other languages interpret it. To anticipate the forthcoming discussion, this alternative way of interpretation is associated with scalar implicatures, possible but not necessary for the speakers of English. In contrast, we will argue that the presence or absence of scalar implicatures enriching the meaning of counterparts of *some* in the other three languages does not affect perceived numerical bounds due to the an additional mechanism of pragmatic strengthening. So far, however, we believe that the point of divergence related to *some* could serve as a basis for a more general evaluation of the nature of quantifiers and a focus on the properties of *some* and its counterparts in the other languages will ultimately shed light on the four questions which motivated this study.

In order to get a clearer understanding of this peculiar difference between *some*, on the one hand and the other existential quantifiers in French, Slovenian, and German, on the other we will look for other patterns of divergence. Recall that the standard semantic-pragmatic theory views the existential quantifier as a trigger of the quantity related implicature. Below we report the results of a second experiment which juxtaposes English and French, the latter as a representative of the group of languages that showed a similar pattern of processing their existential quantifier in Experiment 1.

## Experiment 2

### Implications for the Derivation of Scalar Implicatures

As we saw, there seems to be a difference between English *some* and its counterpart in French, Slovenian, and German. While in the other languages the quantifier is best used for an interval between *a few* and *half*, English *some* is best used for an interval between *a few* and *almost all*. This opens a lot of interesting questions, which have to do with whether this should be seen as a refutation of Grice’s Modified Ockham Razor (in as much as the lexical meaning of the quantifier does not correspond to the logical entailment from *all* to *some*) or as a matter of typicality (see van Tiel, 2014), though the latter possibility would raise the further question of why English would pattern differently from other European languages, including German. But the main question we want to raise here is whether this difference between English *some* and its counterparts impacts the derivation of scalar implicatures. We address this question by comparing English *some* and French *quelques* in a simple picture choice test.

### Experimental Design

We choose a picture choice test paradigm in preference to the more frequently used sentence evaluation task paradigm (see, e.g., Noveck, 2001; Bott and Noveck, 2004) for a number of reasons. Notably, in a sentence evaluation task, the relevant condition is the one where *some* is under-informative, as it is the only one that allows one to differentiate between the pragmatic and the semantic interpretations. However, there are quite a few problems with that task, the first being that the rate of pragmatic answers (which ranges between 40 and 60%) is not clearly different from chance, given that participants have to choose between two answers (putting chance at 50%). This suggests that the infelicity of the experimental condition leads participants to random answers. Another problem is that it is not clear that the task allows a reliable distinction between pragmatic answers (negative) and semantic answers (positive) (see Guasti et al., 2005; Mazzaggio et al., unpublished). Thus, a picture choice task, which offers a reliable distinction between the pragmatic and the semantic answers and avoids the difficulty linked to infelicity seemed by far a better choice. In essence, participants are presented with a sentence with a quantified NP (in the object position) and are asked to choose which among two pictures best corresponds to the sentence. In the *some* condition, one picture illustrates the pragmatic interpretation and the other illustrates the semantic interpretation. We tested French and English native speakers, as we will now describe. To avoid the confound raised by the entailment from *all* to *some*, participants were allowed a single answer. In addition to the *some* experimental condition, we also had an *only some* experimental condition. As Marty and Chemla (2013) have noted, the pragmatic interpretation of *some* has the same content as *only some*, the difference between the two being only the fact that the pragmatic interpretation is implicit. They used thus a comparison between *some* and *only some* and we followed their example.

### Experimental Material

**FIGURE 8:**
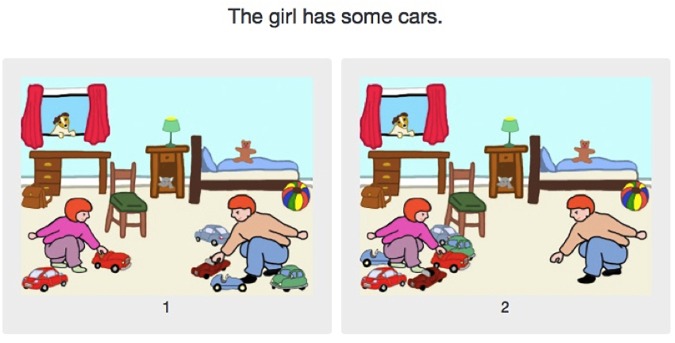
Experiment 2: The *some* condition (pragmatic answer on the left, semantic answer on the right).

**FIGURE 9:**
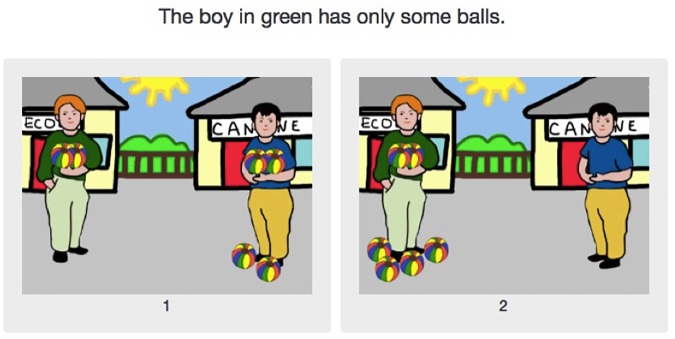
Experiment 2: The *only some* condition (the image verifying the sentence is on the left, the image falsifying it on the right).

**FIGURE 10:**
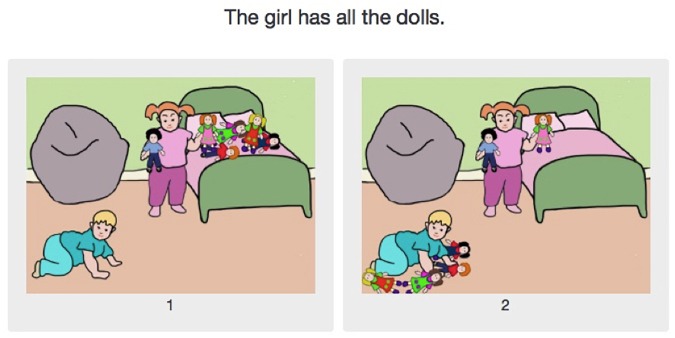
Experiment 2: The *all* condition (the image verifying the sentence is on the left, the image falsifying the sentence in on the left).

The experiment was composed of three main conditions, exemplified in Figure 8–10:

• one control condition, using *all* (four items);
• two test conditions:
  ◦ *only some* (eight items);
  ◦ *some* (eight items);
• Four filler conditions with four items each:
  ◦ *half*;
  ◦ *exactly one*;
  ◦ *exactly two*;
  ◦ *exactly three*.

In the *some* condition, one image corresponds to the pragmatic interpretation and the other to the semantic interpretation, as Figure 8 exemplifies. In all other conditions, including fillers, one image verified the sentence, while the other falsified it. All images presented two characters and six objects, which were either in the possession of a single character or shared among both characters. In the two test conditions (*some* and *only some*), one picture showed the character named in the test sentence with all the objects, while the other showed them with only two of the six objects. Figure 9 exemplifies an example of the other experimental condition, *only some*. As for the control condition, the evaluated sentences contained *all*, as illustrated in Figure 10. The “correct” choice was presented either on the left or on the right in a counterbalanced way. The filler conditions, while not necessarily standardly used in such experiments, also use quantifiers, albeit non-monotonous ones (for *exactly n*), and *half*. As exactly the same fillers were used for the French and for the English groups, it is very unlikely that the choice of fillers had any influence on the wide discrepancy between the French and the English results. Response times were not recorded in Experiment 2, as the question we were interested in was whether the difference between English *some* and French *quelques* evidenced in Experiment 1 would influence the rate of pragmatic answers in this simple picture choice task. It is not clear why response times as such would be directly relevant to that question.

### Participants

Twenty nine French participants were students at the University of Lyon, aged between 18 and 30 (mean age = 21.9; 17 females). They were all native speakers of French. In addition, 34 English participants were recruited through the Prolific platform. They were all students, aged between 18 and 30 (mean age = 23.1; 18 females). There was no significant difference in age across both tested groups (two-tailed *t*-test: *t* = 1.49, *p* = 0.14).

### Procedure

The experiment was presented online on the Qualtrics platform.^9^ It began with a short introduction, where participants indicated sex, age, student status and confirmed that they were native speakers of French or, respectively, English. They were given instructions as well as an example of the task. They then proceeded to the experiment itself. The whole process lasted 10–15 min at the most.

### Results and Discussion

#### Data Treatment and Exclusion

Exclusion was based on more than five items failed in either the control or the filler conditions. No participants were excluded.

#### Response Analysis

**FIGURE 11:**
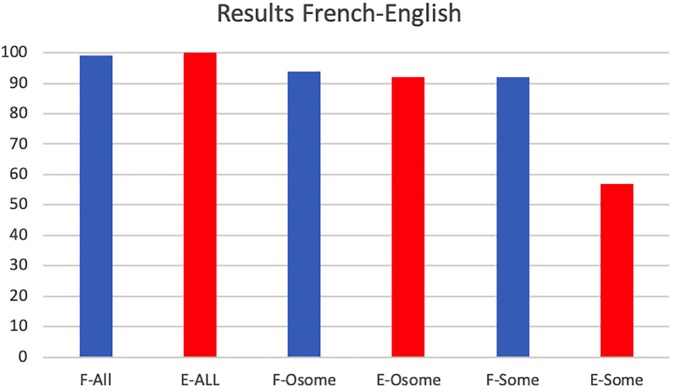
Experiment 2: Response rates when choosing a pragmatic answer by French (blue) and English (red) participants (E, English; F, French; All, the *all* condition; Osome, the *only some* condition; *Some*, the *some* condition).

The rates of response in choosing a pragmatic answer are summarized in Figure 11. Comparing the response rates of choosing a pragmatic interpretation in English and French, we find that French and English participants behave similarly in the *all* control condition choosing pragmatic answers in virtually all cases (French: 99.13%, English: 100%; χ^2^ test: χ^2^(1) = 0.002, *p* = 0.96, no significant difference at the 0.05 level) and in the *only some* test condition (French: 94.39%, English: 92.64%; χ^2^(1) = 0.021, *p* = 0.88). However, they behaved very differently in the *some* test condition, whereby French participants chose the pragmatic interpretation at a higher rate than did the English participants (French: 92.24%, English: 57.30%; χ^2^(1) = 11.901, *p* = 0.0005) and at a rate similar to that of their interpretation of *only some*. Correspondingly, French participants did not differ in their response rate on *some* and *only some* conditions [χ^2^(1) = 0.029, *p* = 0.86], while English participants showed a significantly greater preference for the targeted answer on the *only some* condition compared to the *some* condition [χ^2^(1) = 13.9040, *p* = 0.0003].

#### Discussion

We tested French and English participants in the simple image-choice task for three main conditions: an *all* control condition, a target *only some* condition, and a target *some* condition. This last condition was intended to establish whether French and English participants draw the scalar implicature at the same rate despite the difference in the interval inside which, respectively, *quelques* and *some* are best used in the two languages. It appears that they do not.

## General Discussion

There are two general patterns that emerge from the cross-linguistic studies we report. The first one is that French, Slovenian and German counterparts of *few*, *half*, *most*, and *almost* are assigned very similar numerical bounds. The second one is that *some* and its variants like *quelques* are different in more than one way, namely: (i) with respect to numerical bounds, and (ii) with respect to their potential to trigger a scalar implicature.

In what follows we attempt to account for these facts by arguing that the set of quantifiers viewed as a natural class by the standard semantic-pragmatic theory is, in fact, diverse and the set-theoretic semantics is not appropriate for all of its members. As a consequence, the mechanism of pragmatic enrichment that these items trigger is of a different nature. Finally, we will argue that it is possible that languages do not assign the same kind of semantic definition to determiners that might, from a cross-linguistic perspective, look like translational equivalents.

We start with the point that not all quantifiers quantify over sets of individuals. There have been numerous proposals in the semantic literature that argue against the standard set-theoretic analysis and in favor of a degree-based analysis for some items. Classical cases involve *most*, *many*, *much*, *few*, *half* (cf. Rett, 2008, 2015; Hackl, 2009; Solt, 2009, 2015, etc.). However, if we assume that these particular quantifiers have a different semantic nature, we might face a challenge in restricting the application of those pragmatic principles which rely on the availability of semantic alternatives that constitute a natural class. Crucially, this affects the derivation of quantity-based implicatures. Of course, all determiners that we have considered so far, including the ones we did not test like *every*, *all*, *no*, etc. have the same brevity, or roughly, morphological complexity and thus satisfy the basic criterion for serving as a source of a scale of alternatives (cf. Levinson, 1983, 2000). However, a stricter requirement on their semantic make-up can leave some of these determiners outside of the set of possible alternatives to *all/every*, for example. But even if this is so, the results from our Experiment 1, as well as the results from the other psychometric and typicality-based studies indicate that the meanings of degree-based determiners are pragmatically enriched because they differ from the respective truth-conditional meanings. So if quantity implicatures are not always available for pragmatic enrichment in the domain of quantifiers, how can one account for pragmatic strengthening in all degree-based quantifiers?

A possible answer comes from a proposal in Stateva and Stepanov (2017). That proposal extends Krifka’s (2007a) analysis of negated antonyms (like *happy*, *not happy*, *unhappy*, *not unhappy*) to the domain of the Slovenian degree quantifiers *precej* and *veliko*, both of which are counterparts of the English *many*. The gist of the proposal is that *precej* and *veliko* are semantically equivalent but their meanings are differentiated as a result of pragmatic enrichment through an M-implicature and an R/I-implicature, respectively. R/I-implicatures are associated with a stereotypical interpretation while M-implicatures are related to non-stereotypical interpretations (Horn, 1984; Levinson, 2000). A prerequisite for the Krifka-type analysis is a state of affairs in which there is at least one pair of antonyms so that together they exhaust a relevant degree scale as contradictories. Since degree predicates are vague, the cut-off point is related to epistemic uncertainty for the speaker (Williamson, 1994). In the availability of synonyms in the positive or the negative extension of the scale, as is the case with the two Slovenian positive amount words *precej* and *veliko* that are antonyms to the negative *malo* “few,” a stereotypical interpretation, i.e., an interpretation which is related to a segment of the positive scale which is at a safe distance from the potential cut-off point is assigned to one of the synonyms as an R/I-implicature. The stereotypical interpretation is then always closer to the endpoint of the scale than the non-stereotypical interpretation which results from the application of an M-implicature. If we generalize on the basis of the Slovenian case involving the two quantifiers *precej* and *veliko*, we will have a potential mechanism of pragmatic enrichment of other degree quantifiers which are part of a paradigm that contains at least one antonym and at least one synonym to them.

Very importantly, the above suggestion does not exclude quantity implicatures in the degree domain in general. Under a strict version of restricting scalar alternatives, we expect that, for example, *most* and *all* should not be members of a Horn-set given that one involves quantification over degrees and the other, quantification over individuals but the two degree-based quantifiers *most* and *almost all* would. This would explain why the upper part of the degree scale is not accessible for *most* (although the truth-conditional meaning of *most* is compatible with it). Arguably, *most* triggers a scalar implicature that negates the *almost-all* alternative.

We now have the ingredients for a proposal that explains the facts from the reported experiments. We would like to suggest that the existential quantifiers that we tested are of different semantic nature and because of that they are subject to different processes of pragmatic strengthening. To English *some* we attribute the standard semantic meaning as relating two sets of individuals. The results from both Experiment 1 and Experiment 2 suggest that *some* is pragmatically enriched with a scalar implicature. This hypothesis is confirmed (i) by the larger acceptability interval on the proportion scale for *some* in comparison to the rest of the tested existential quantifiers where *some* covers also very high ratios, as predicted, and (ii) by the lower rate of scalar implicature derivation associated with *some* in comparison to *quelques* which is also expected given the optional character of scalar implicatures. As for the counterparts of *some* in French, Slovenian, and German, we would like to suggest that they are degree-based quantifiers, lexically synonymous to the lexical items corresponding to *few* in each of the languages and antonyms of the lexical items corresponding to *many*. In this analysis, the French *quelques*, Slovenian *nekaj* and German *einige* are associated with the lower part of the degree scale while the respective counterparts of *many* in each language are associated with intervals above the cut-off point. All three languages have a lexical version of *few* which competes with *quelques*, *einige*, or *nekaj* for the stereotypical or non-stereotypical interpretation. Our results from Experiment 1 suggest that *quelques*, *einige*, and *nekaj* are pragmatically enriched with the non-stereotypical implicature and are thus at a greater distance from the scale and-point in comparison to the stereotypically interpreted counterpart of *few*. Some overlap within synonym pairs in each of the languages is always expected due to epistemic uncertainly because of the vague character of quantifiers that do not denote end points. In much the same vein in which speakers are uncertain about the cut-off point on a relevant scale between two antonyms and simultaneously have a whole set of potential cut-off points under consideration speakers entertain a set of cut-off points within the scale part associated with the pair of synonyms. As a result of epistemic uncertainty, there are overlaps in all zones coinciding with potential points of delineation.

This explanation gets further support from the results of Experiment 2. Recall that in the Picture-Choice task, French speakers were at ceiling with the choice of the pragmatic meaning while English speakers had a significantly lower rate of choosing the pragmatic answer in comparison to the French speakers. These facts are consistent with the hypothesis that the pragmatically enriched English target sentence results from a scalar implicature negating the *all*-alternative which is only optional (Chierchia, 2013). When the implicature is forced by the explicit use of *only*, speakers responded in accord with expectations and performed at ceiling, too. The relevant pragmatic alternative for the French speakers in the target condition is, in fact, not based on *all* but rather on *few* and so the non-targeted answer did not interfere in this case.

The proposal makes a prediction for the relation between *many* and *some* and their respective counterparts. These items are a pair of antonyms in French/Slovenian/German-type languages. This entails some overlap region on the degree scale corresponding to the zone where different cut-off points are under consideration because of epistemic uncertainty but the overlap cannot be too large. As for English, *some* and *many* can partially overlap to a greater extent. We have indirect confirmation of this prediction from Experiment 1: as we saw previously, unlike its counterparts, English *some* is acceptable in contexts with very high proportions bordering the region reserved for the upper part of *most* and *almost all*. This interval can be reasonably expected to contain the interval allotted to *many*.

This is the stronger version of the proposal we want to push forward. A weaker version of it would not exclude scalar implicatures based on entailment relations even among the members of the class of quantifiers that are triggers of R/I-implicatures or M-implicatures in French/Slovenian/German, i.e., among the counterparts of *some*, *few*, and *many*. To give substance to this possibility we can refer to Chemla (2007) and Buccola et al. (2018) which suggest that scales of alternatives are based on concepts rather than lexical elements. If this is so, a Horn-set of alternatives can well be formed by quantifiers that do not denote functions of the same semantic type. Under this weaker proposal, however, the availability of M-implicature for the French, Slovenian and German counterparts of *some* in contrast to the R/I-implicature triggered by the counterparts of *few* would trivialize the effect of quantity induced implicatures which, in this case, would not be necessary to explain the facts about the existential quantifier cross-linguistic differences we observe in Experiments 1 and 2.

The results from Experiment 1 are in line with the findings of Pezzelle et al. (2018). They demonstrate that quantifiers are perceived as part of an ordered scale which involves overlaps (i.e., similarities) of different dimensions. We argued that quantifier differentiation depends on more than one mechanism of pragmatic enrichment on a par with the semantic makeup of quantifiers as potential alternatives.

As we stated above, our research question about quantifier meanings in language use is focused on potential cross-linguistic variation. The data we collected suggests that such differences exist and they have a systematic character as confirmed by both experiments reported here. Let us, however, consider briefly some other studies bearing on cross-linguistic variation among quantifiers. Katsos et al. (2016) reports a study on quantifier acquisition by 5-year-old children in 31 languages, among which three of the languages discussed here: English, French, and German. That study includes a task on the counterparts of *some* and *most* which makes the comparison between both studies possible. However, the German existential quantifier tested in Katsos et al. (2016) is *ein Paar* and since it is different from *einige* used in our study, we will limit our attention to English and French only. In contrast to our study, Katsos et al. (2016) does not report any relevant cross-linguistic variation. Whether this result contradicts the results we report, however, can only be appreciated if we scrutinize the research questions and the tested hypotheses of that study. Katsos et al. (2016) investigate whether the order of acquisition of quantifiers is similar across languages given a number of factors related to the formal properties of different determiners. More specifically, these are monotonicity (upward vs. downward), totality [related to scale endpoint (e.g., *no*, *all*) or non-end-point (*some*, *most*)] (morphological) complexity and finally, truth versus felicitousness, i.e., whether pragmatic meaning is acquired after semantic meaning.^10^ In effect, the comparisons track the acquisition order among quantifiers within each language but not the order of acquisition of translational equivalents among languages which would have been indicative of potential cross-linguistic differences among translational equivalents. In particular, results reported from testing 17 English speaking children and 15 French speaking children show that in both languages, accuracy of *some*/*quelques* is higher in comparison to *most*/*la plupart*, respectively, as predicted by the hypothesis that *most*/*la plupart*, being the superlative form of *many*, is morphologically more complex than *some*/*quelques*. However, this finding is orthogonal to our study because there is no a priory reason to assume that a quantifier based on degrees, as we argue, is more complex, and therefore, more difficult to acquire than a quantifier that relates sets of individuals. Consequently, the comparable accuracy of English and French participants on the conditions related to the acquisition of *some* in English and *quelques* in French, respectively, cannot be interpreted as a counterargument against the proposal we are advancing. What is more, the data obtained by Katsos et al. (2016) is not inconsistent with the data obtained within the study we report.

Before we conclude this discussion, we would like to mention two facts that could serve as independent evidence for our proposal. The first one is based on an observation about the morphological makeup of the plural morphology paradigm in Bulgarian. Bulgarian features two plural nominal agreement patterns in the masculine paradigm. The default case is a plural ending that agrees with the plural morpheme of any adjectival modifier within the nominal phrase. The second one, known as the “count form,” is non-agreeing, and is selected if the noun is preceded by a numeral (cf. Stoyanov, 1980; Stateva and Stepanov, 2016, etc.). Both plural patterns are exemplified in (2a) in (2b), respectively:

(2) a. Červen-i (dârven-i) prozorec-i
  red-pl wooden-pl window-pl
  “red (wooden) windows”

      b. Pet (dârven-i) prozorec-a
         five wooden-pl window-count
         “five (wooden) windows”

Interestingly, the count form is also used when the noun contains the existential quantifier *njakolko* “some” but not when it contains the universal one *vsichki* “all,” as shown in (3):

(3) a. Njakolko (dârven-i) prozorec-a/^∗^prozorec-i
  some wooden-pl window-count/window-pl
  “some wooden windows”

      b. Vsichki (dârven-i) prozorec-i/^∗^prozorec-a
         all wooden-pl window-pl/window-count
         “all wooden windows”

The parallel between numerals and *njakolko* indicates that they belong to the same natural class to the exclusion of *vsichki*. The possibility of having a numeral-like existential quantifier in one language suggests a similar possibility for other languages even in the absence of morphological makeup indicative of the specific semantic nature of the quantifier.

Second, our proposal can account for the observation that numerical bounds of vague quantifiers depend on the cardinality of the total set. If pragmatic enrichment of degree-based quantifiers that come in pairs of antonyms and synonyms depends on delineation between lower and upper scale parts, as well as on interval assignment to stereotypical and non-stereotypical, we can expect that partitioning in a closed scale of this kind will involve a lot of overlaps. This is so because each interval to which a quantifier is related in this case ends up being too small to be distinguished from the neighboring ones, especially in view of epistemic uncertainty. It follows then that different numerical bounds are associated with the same quantifier in small and larger sets where competing alternative quantifiers are assigned to greater scale intervals.

## Conclusion

We conclude by going back to the questions we posed in the Section “The Present Study.” We started with the question of whether it is possible to identify the different quantifiers’ numerical bounds and whether these are encoded in quantifier meanings or are epiphenomenal. The answer that follows from our discussion is that numerical ranges are epiphenomenal: they result from pragmatic strengthening and no additional meaning component needs to be postulated in order to account for the difference between lexical meanings and actual judgments in tasks.

We believe that the cross-linguistic perspective that we added to this study sheds light on the question of whether quantifier meanings can be given the status of a semantic universal (Determiner Universal, Barwise and Cooper, 1981). If our interpretation is correct, the existential quantifier is a source of considerable cross-linguistic variation. An anonymous reviewer raises a question related to it. It pertains to the source of this difference from the point of view of language change. While it is not that difficult to assume that a Slavic language like Slovenian, or a Romance language like French might differ in some fundamental aspect from English, which belongs to the Germanic family, it is much less obvious why English and German would not pattern together. Assuming that there has been a common source for the existential quantifier, it is important to look for an answer to the question about the trigger of the semantic shift and the trajectory leading to these two different patterns. While we acknowledge the importance of this question, it falls beyond the focus of our current study and therefore we leave it for future research.

We identified two types of pragmatic enrichment processes that are operative in the domain of quantifiers: quantity-based enrichment through scalar implicatures, and stereotypical and non-stereotypical meaning enrichment through R/I-implicatures and M-implicatures. We argued that if we assume a cognitive-based definition of pragmatic alternative, both processes are operative but in some cases the effect of quantity induced implicatures is trivialized.

Finally, we come to the question of overlapping meanings. We argued that meaning overlap is language dependent and is less likely to be expected in cases that involve pragmatic strengthening through R/I- and M-implicatures.

## Ethics Statement

The experiments in this study were carried out in accordance with the Declaration of Helsinki and the existing European and international regulations concerning ethics in research. All participants gave an informed consent prior to the beginning of testing. In addition, the English portion of Experiment 1 was approved by the IRB at Rutgers, the State University of New Jersey. Experiment 2 was carried out in accordance with the recommendations of the Comité de Protection des Personnes Sud Est II, who gave it its agreement (IRB number: 11263).

## Author Contributions

PS and AS designed the Experiment 1 and collected the Slovenian data. PS participated in collecting the German data. LD and VD collected the French and English data, respectively, for Experiment 1. AR designed the Experiment 2 and collected the French and English data for it. PS, AS, and AR analyzed all of the data and wrote the manuscript.

## Conflict of Interest Statement

The authors declare that the research was conducted in the absence of any commercial or financial relationships that could be construed as a potential conflict of interest.

## Footnotes

1 The German data were collected by Stateva and Gergel for a study published as Gergel and Stateva (2014) focusing on the differences between the German determiners *allermeisten* (“most”) and *fast alle* (“almost all”). Except for *fast alle*, the data collected on the German quantifiers as in Table 1 were not discussed in that study and were largely treated there as filler conditions.

2 We avoided the use of round numbers in the contexts for two reasons. First, we wanted the participants not to be tempted to provide judgments with the help of explicit ratio calculations. Second, since it has been shown that round numbers invite more approximate interpretations (Krifka, 2007b), we believed that by avoiding round numbers, we prompt as precise interpretations as possible, especially in view of the task to evaluate vague quantifiers.

3 Scores were reassigned as follows for the analysis: 1→5, 2→4, 4→2 and 5→1.

4 All simulations and modeling in this study were performed in the R environment 3.5.1 (R Core Team, 2018).

5 https://www.soscisurvey.de/

6 Standard protocols in experimental pragmatics like the Truth Value Task or Felicity Judgment Task involve evaluation of alternatives on the basis of pragmatic reasoning restricted by pragmatic principles like Quantity (Grice, 1989), Maximize Presupposition (Heim, 1991), etc. This methodology allows for testing implicated meaning which is also relevant to quantifier comprehension.

7 As the experiment also probes into pragmatic knowledge about quantifier meaning, we need to acknowledge the paramount importance of the methodology of judgment collection. Appealing to a pragmatic tolerance principle that potentially obscures existing sensitivity in binary tasks, Katsos and Bishop (2011) argue against the elicitation of a binary judgment in developmental pragmatics (but see also Noveck, 2018). The non-binary judgment approach fits our research question better. Given that we expect some degree of similarity in the perception of different quantifiers, we believe that considering an interval of acceptable points, rather than a point of acceptability is the more informative choice.

8 We stress, in this regard, that all observed correlations were *post hoc* and as such could not have affected our choice of the 3-point acceptability threshold itself (this thresholding methodology was also used in a similar experiment reported in Stateva and Stepanov, 2017).

9 http://www.qualtrics.com

10 Upward monotone quantifiers license inferences to supersets while downward monotone quantifiers license inferences to subsets.
